# Sensitivity analysis of a reduced model of thrombosis under flow: Roles of Factor IX, Factor XI, and γ‘-Fibrin

**DOI:** 10.1371/journal.pone.0260366

**Published:** 2021-11-23

**Authors:** Jason Chen, Scott L. Diamond

**Affiliations:** Department of Chemical and Biomolecular Engineering, Institute for Medicine and Engineering, University of Pennsylvania, Philadelphia, PA, United States of America; Institute of Experimental Hematology and Transfusion Medicine, University Clinic of Bonn, GERMANY

## Abstract

A highly reduced extrinsic pathway coagulation model (8 ODEs) under flow considered a thin 15-micron platelet layer where transport limitations were largely negligible (except for fibrinogen) and where cofactors (FVIIa, FV, FVIII) were not rate-limiting. By including thrombin feedback activation of FXI and the antithrombin-I activities of fibrin, the model accurately simulated measured fibrin formation and thrombin fluxes. Using this reduced model, we conducted 10,000 Monte Carlo (MC) simulations for ±50% variation of 5 plasma zymogens and 2 fibrin binding sites for thrombin. A sensitivity analysis of zymogen concentrations indicated that FIX activity most influenced thrombin generation, a result expected from hemophilia A and B. Averaging all MC simulations confirmed both the mean and standard deviation of measured fibrin generation on 1 tissue factor (TF) molecule per μm^2^. Across all simulations, free thrombin in the layer ranged from 20 to 300 nM (mean: 50 nM). The top 2% of simulations that produced maximal fibrin were dominated by conditions with low antithrombin-I activity (decreased weak and strong sites) and high FIX concentration. In contrast, the bottom 2% of simulations that produced minimal fibrin were dominated by low FIX and FX. The percent reduction of fibrin by an ideal FXIa inhibitor (FXI = 0) ranged from 71% fibrin reduction in the top 2% of MC simulations to only 34% fibrin reduction in the bottom 2% of MC simulations. Thus, the antithrombotic potency of FXIa inhibitors may vary depending on normal ranges of zymogen concentrations. This reduced model allowed efficient multivariable sensitivity analysis.

## Introduction

Blood clotting occurs under flow conditions in many circumstances of hemostasis or intravascular thrombosis. When tissue factor is exposed to the blood the coagulation cascade is triggered, resulting in the eventual generation of thrombin and the polymerization of fibrin. The molecular events of this protease cascade are well studied and computer simulations of isotropic coagulation (TF added to plasma) typically include 20 to 60 individual parameterized reactions [1–3]. The complexity is increased by the presence of flow, the participation of platelets in clot growth, and various strong couplings and feedbacks [4–6]. Detailed models of coagulation under flow often require 20 to 50 partial differential equations (PDEs) and about 100 parameters relating to initial concentrations and kinetic coefficients. Numerous reviews have discussed both continuum and particle-based numerical approaches [7]. The further goal of multiscale modeling seeks to deploy complex vascular flows with realistic models of platelet signaling and coagulation function [8–10], all of which is extremely demanding from a computation point of view. Reduced models offer advantages in bridging scales and in handing 3D coupled reaction-diffusion-convection problems.

For clotting isotropically in a tube or clotting under flow conditions, plasma zymogen variations can be studied by Monte Carlo simulation or with high throughput experiment to explore sensitivity to initial conditions [2]. Blood plasma contains zymogens whose concentrations vary in the healthy population [11]. These variations impact coagulation time as was seen in a sensitivity analysis that highlighted the most proximal FVIIa participating reactions [12]. Often, the time to generate thrombin is the key parameter used in sensitivity analysis [13]. For example, using a sensitivity analysis of a thin film compartment model of clotting under flow, Leiderman et al. identified an unexpected competitive reaction involving Factor V that influenced hemophilic severity [14]. However, these models often have reaction networks that do not include the dynamics of fibrin generation and the binding of thrombin to fibrin via a weak site and a strong site. Fibrin has ‘antithrombin-I activity’ which includes (i) thrombin exosite I binding to the low affinity (Kd~2.8 μM site) in the E Domain, (ii) thrombin exosite II binding the high affinity site (Kd~0.1 μM) in the D-domain of the alternative splice variant, γ’-fibrin(ogen), (iii) a potential bivalent interaction, and (iv) irreversible entrapment [15]. The γ’-fibrinogen splice variant represents about 6–8% of total γ’-chains, with γA/γ’ heterodimer representing 12–16% of total fibrinogen [16].

Typically, simulating reactions under flow requires PDE models, however the thin film approach can reduce the system to ordinary differential equations (ODEs) that include mass transfer coefficients [17] or accommodate transport limits with effectiveness factors (η = actual rate/ideal rate) [18]. In the present study, we explored the sensitivity of an extrinsic pathway model for a 15-micron film that was previously shown to simulate fibrin formation dynamics and thrombin generation dynamics.

## Methods

**Fig 1:**
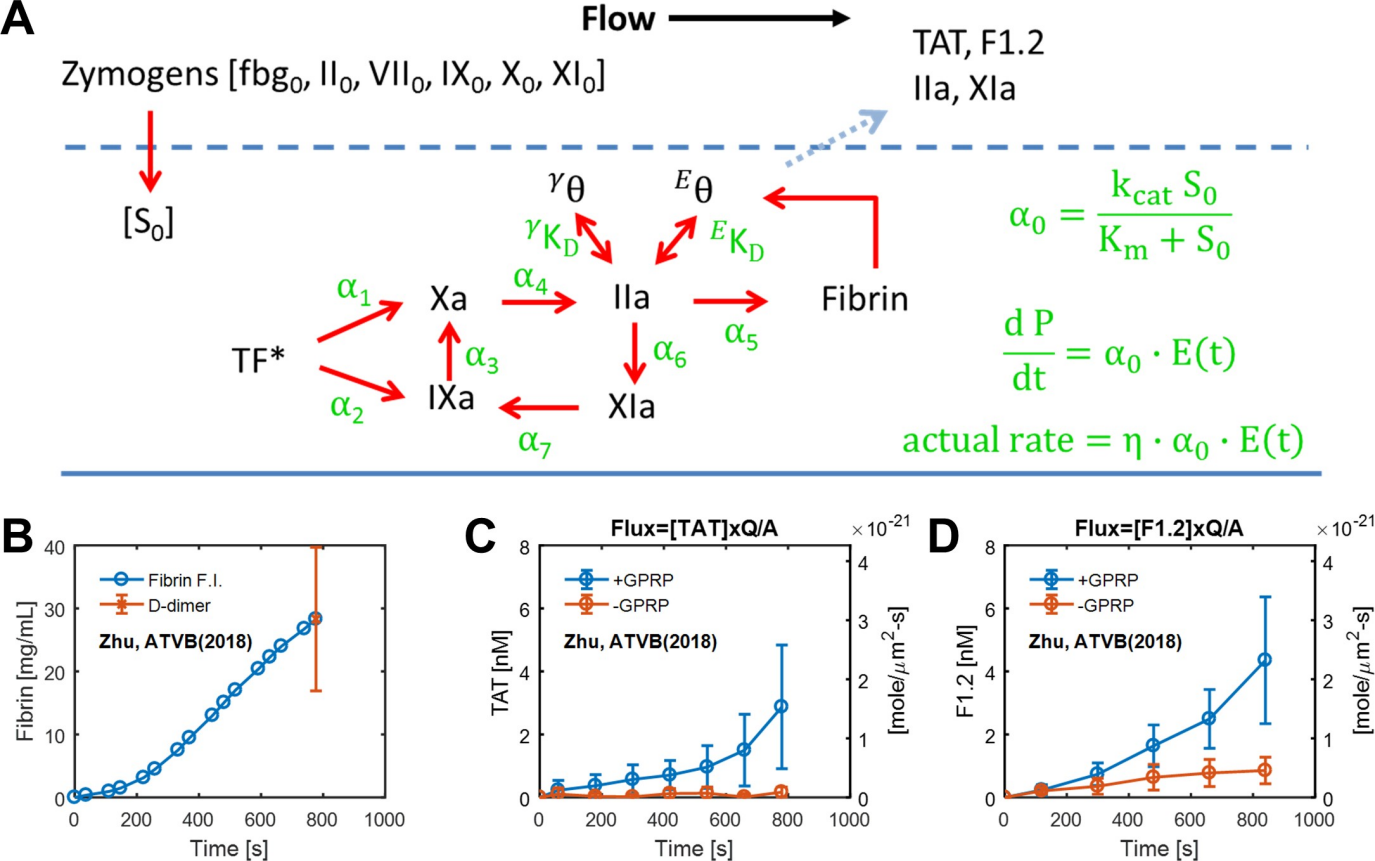
Schematic of the reduced model. With 7 reactions, only the activated proteases are shown (A). All zymogens were assumed to enter the clot core by diffusion to maintain their plasma level [S]_o_. All active enzymes had a 1-minute half-life, with TF* set to 3 min. Elution rate from the core was set to 2-sec half-life. The dynamic of fibrin from 8-channel device of the fibrin fluorescence intensity with the end-point concentration determined by D-dimer ELISA (B). Thrombin flux from the collagen/TF surface determined by thrombin-antithrombin complex (TAT) ELISA with and without GPRP to allow fibrin formation (C). Thrombin flux from the collagen/TF surface determined by fragment F1.2 ELISA with and without GPRP to allow fibrin formation (D).

The reduced model supported by experimental data to predict thrombosis under flow was described previously [18]. Briefly, the model simulates blood clotting under venous flow over collagen and TF (1 TF/ μm^2^) with a 15-micron thick “core” region (δ = 15 μm, porosity ~0.5), features supported by direct imaging and experimental configuration. The effective volume for enzyme reaction and initial conditions are shown in **S1 File**. The blood was treated with high CTI (40 μg/mL) to inhibit FXIIa as discussed in [18,19] so the model does not include FXIIa. We have previously shown CTI at this concentration prevents the formation of F1.2 when whole blood is perfused through a device lacking collagen/TF. The model includes extrinsic tenase/FIXase activity, intrinsic tenase activity, prothrombinase activity, feedback activation of FXIa by thrombin, fibrin generation, and thrombin bindings to fibrin. The thin film assumption allowed the linearization of the Michaelis-Menton kinetics since zymogen concentrations are set constant to plasma levels. The reduced model (7 rates, 2 K_D_ values, and enzyme half-lives ~ 1 min) only required 3 adjustments from published values measured under static conditions to predict the elution rate of thrombin-antithrombin (TAT), fragment F1.2 with or without fibrin formation, and intrathrombus fibrin. For the clot effluent measurements of TAT where thrombin had time to be inhibited, 70% of the thrombin eluted from the clot was considered complexed with antithrombin with the remaining 30% of eluted thrombin complexed with other inhibitors [19]. The schematic of the reduced model is shown in **Fig 1A**. The experimental data of fibrin dynamics and F1.2/ TAT flux with/without fibrin formation are shown in **Fig 1B–1D**. The simulation results compared to experimental data are shown in **S1 File**.

The initial concentration of 5 plasma zymogens (FXI, FIX, FX, FII, fibrinogen) and 2 fibrin binding sites (weak sites and γ’-sites) are obtained from literature value and shown in **Table 1**. The results for population-average initial concentrations on coagulation rates are shown in **S1 File**, including the dynamics of FIXa, FXa, FXIa, thrombin on different sites, Fibrin, and F1.2/ TAT flux. Sensitivity analysis evaluate the effect of each variable on model predictions. We followed a similar sensitivity analysis used by a recent study [20]. Although there are several ways for sampling [12], we varied the concentration of 7 variables (5 zymogens and 2 thrombin binding sites) ± 50% as described in [20]. First, we performed a local sensitivity test where the variables were changed one-at-a-time. For visualization, the change of the maximum concentration of each species over 800 sec, instead of whole dynamics, was shown with respect to a range of variant. We then performed 10,000 Monte Carlo simulations where all the variables were sample uniformly and independently between 50–150% from their baseline concentration.

**Table 1:** Reactions and kinetic parameters used in the ODEs model.

#	Reactions	Enzyme	[S]_0_	k_cat_ (s^-1^)	K_m_ (μM)	Α (s^-1^)	η	Ref.
1	$X\overset{{FVIIa}^{*}}{\to }Xa$	TF/VIIa	X_0_ = 0.17 μM	1.15	0.24	0.46	1	[21]
2	$IX\overset{{FVIIa}^{*}}{\to }IXa$	TF/VIIa	IX_0_ = 0.09 μM	1.8	0.42	0.32	1	[2]
3	$X\overset{IXa}{\to }Xa$	IXa/VIIIa	X_0_ = 0.17 μM	8.2	0.082	5.42	1	[2]
4	$II\overset{Xa}{\to }IIa$	Xa/Va	II_0_ = 1.4 μM	30	0.3	24.7	0.18	[2,21,22]
5	$II\overset{Xa}{\to }IIa$	IIa	α-fbg_0_ = 18 μM	80	6.5	5.88	0.05	[2,23]
6	$XI\overset{IIa}{\to }XIa$	IIa/p*	XI_0_ = 31 nM	1.3x10^-4^	0.05	4.98x10^-5^	0.36	[2]
7	$IX\overset{XIa}{\to }IXa$	XIa/p*	IX_0_ = 0.09 μM	0.21	0.2	0.065	1	[2,24]
	**thrombin binding to fibrin**			**K**_**d**_ **(μM)**	**k**_**f**_ **(μM**^**-1**^**s**^**-1**^**)**	**k**_**r**_ **(s**^**-1**^**)**		
1	IIa+E site↔IIa∙E site			2.8	100	280		[25]
2	IIa+γ site↔IIa∙γ site			0.1	100	10		[25]

Simplified clotting reactions neglecting limits in activated cofactor generation, plasma zymogen concentrations, and kinetic parameters of coagulation where η is the effectiveness factor (actual rate with transport limits/theoretical maximum rate). For each reaction, α_o_ = k_cat_ [S]_o_/(K_m_+[S]_o_). Published kinetic parameters above were typically obtained with excess purified lipids containing phosphatidylcholine (PC) and phosphatidylserine (PS) as a laboratory mimic of the more biologically complex platelet membranes. Reversible binding of thrombin to the weak and strong site in fibrin was treated as kinetically-controlled, reversible adsorption. [18] (p*, activated platelet).

The dynamics of fibrin deposition (calibrated by D-dimer assay) in 8-channel devices has been used to evaluate coagulation in whole blood under flow [26–29]. Therefore, we marked the top and bottom 2% of the fibrin concentration and looked at the distribution of the 7 variables. To evaluate the potency of a perfect FXIa/FXI inhibitor, we can turn off the feedback pathway described in [18] by setting α_6_ and α_7_ (in **Table 1**, **S1 File**) to zero.

## Results

### Local sensitivity analysis

By thin film assumption, we were able to simplify the perfusion of CTI-treated (40 μg/mL, full FXIIa inhibition without effect on FXIa) whole blood clotting over collagen/ tissue factor surface (250 μm, 1 TF/ μm^2^). Only 3 parameters were adjusted from literature to fit the measured TAT, F1.2 and fibrin generation data (± GPRP). The reactions and kinetic parameters used in the reduced model are shown in the **Table 1**. In Table 1, reactions 1,2,3, and 7 displayed no transport limits (effectiveness factor = 1), while reactions 4,5, and 6 were adjusted to fit the experimental data, consistent with the presence of transport limits (effectiveness factor < 1). Thus, only 3 parameters were adjusted from literature (η4,5,6 < 1) to fit the measured TAT, F1.2 and fibrin generation data (± GPRP), which could indicate the transport limits (especially for fibrinogen) or possible differences due to conditions on platelet membranes of whole blood experiments. With well mixed, substrate concentrations in the clot are set to be constant at plasma levels from literatures. The dynamics of the procoagulants, thrombin distribution, fibrin, flux of F1.2 and TAT predicted by the reduced model with the baseline of plasma protein levels and thrombin binding sites are shown in **S1 File**.

**Fig 2:**
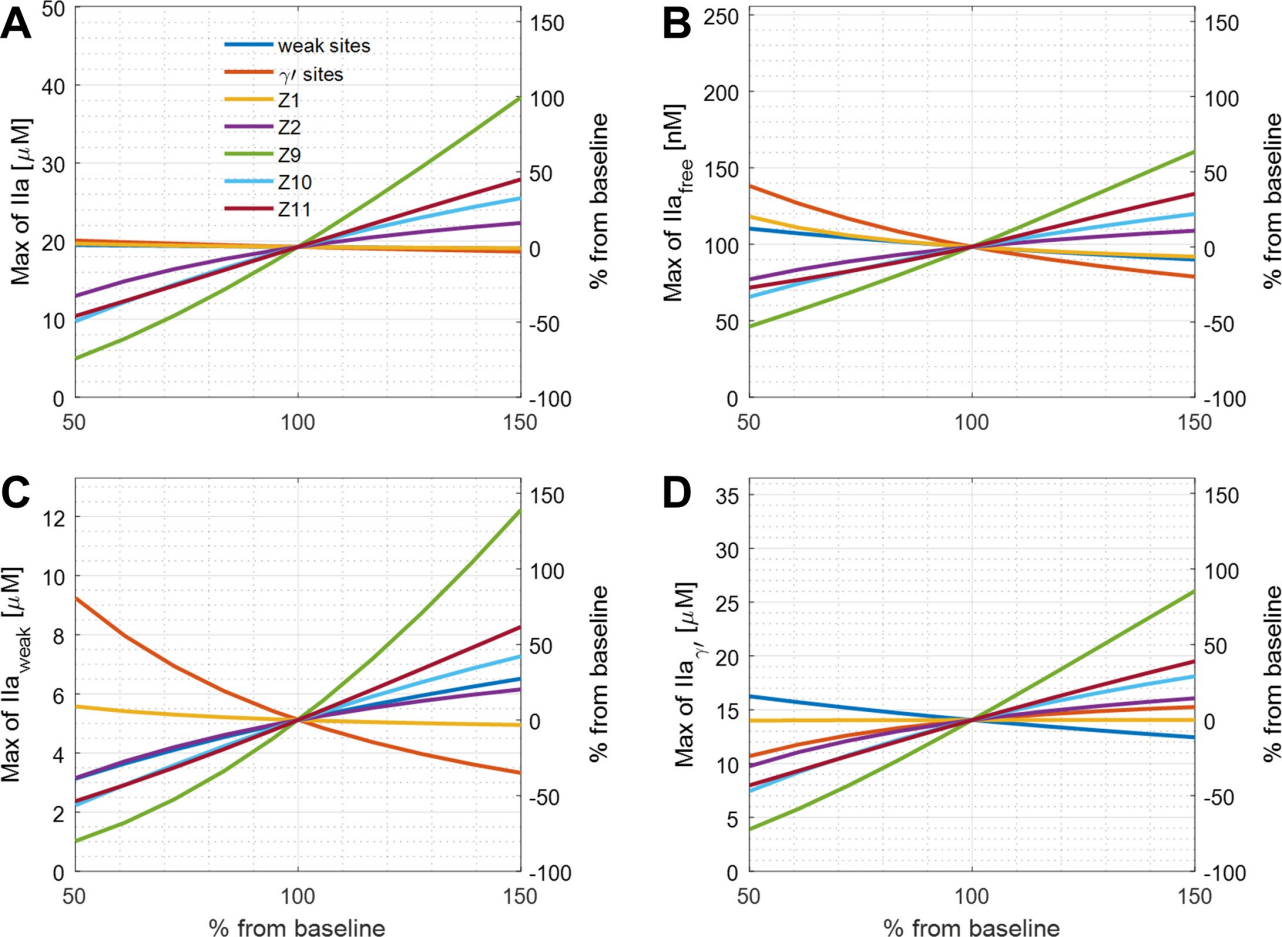
Local sensitivity analysis of total thrombin, free, and bound thrombin in the clot. The change of intrathrombus thrombin concentration on different sites due to the variation of plasma protein levels or thrombin binding sites on fibrin. The levels were changed one-at-a-time.

**Fig 3:**
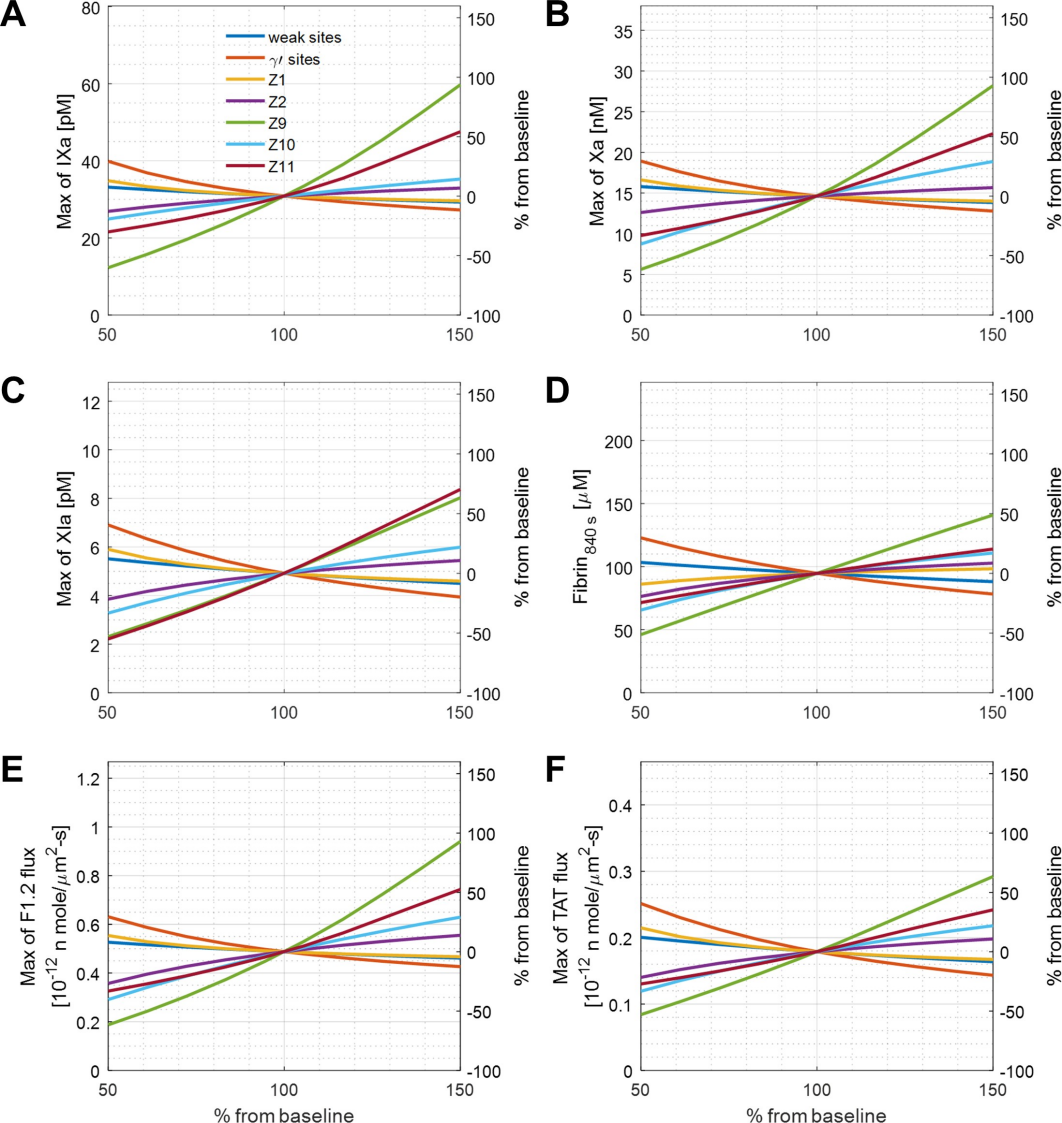
Local sensitivity analysis of procoagulants, fibrin, F1.2 flux, and TAT flux. The concentration change of FIXa (A), FXa (B), FXIa (C), fibrin (D), and flux of F1.2 (E) and TAT(F) due to the variation of plasma protein levels or thrombin binding sites on fibrin. The levels were changed one-at-a-time.

Importantly, the levels of plasma zymogens and fibrin binding sites for thrombin vary across a range in healthy individuals. Here, we analyze the sensitivity of the reduced model output of all species (FIXa, FXIa, FXa, thrombin on different sites, TAT/F1.2 flux and fibrin). We first used the method of changing a variant one-at-a-time to quantify the sensitivity of each output, which was used by [20]. We changed a variant one-at-a-time at a range of 50% to 150% of normal with others fixed. The local sensitivity of thrombin concentration on different sites of fibrin are shown in **Fig 2**. The results suggest that FIX level is the most important factor for thrombin. When FIX level is increased 50% from the baseline, total thrombin and thrombin binding on to weak sites increase by 100% and 140%, and free thrombin and thrombin on γ’-sites increase by around 75% compared to baseline. Maximum thrombin concentration is less sensitive to the variation on both thrombin binding sites. A stronger effect was seen when the γ’-sites decreased by 50%, thrombin on weak sites and free thrombin increased by 80% and 40%. The variation leads to similar trend on procoagulant factors (FIXa, FXa, FXIa), and the results are shown on **Fig 3**. Variation in FXI has more effect on FXIa. Fibrin as the final product of the coagulation cascade did not change dramatically with the zymogen variations. The fibrin concentration stays within ± 50% by varying each inputs. Lastly, there were similar effects on F1.2 and TAT flux (**Fig 3E and 3F**), but the effect on F1.2 was stronger.

### Global sensitivity test

**Fig 4:**
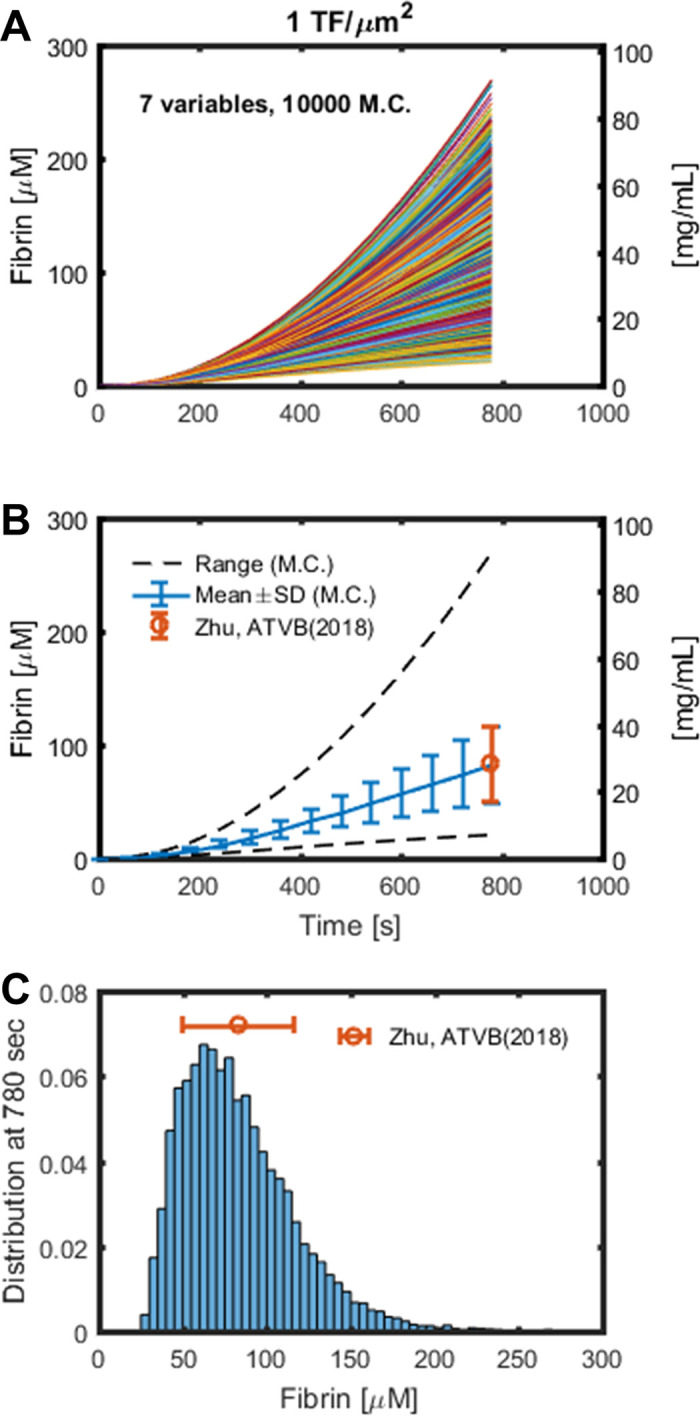
Fibrin concentration of 10,000 simulations compared to experimental data of blood clotting over collagen/ TF under venous flow rate. Fibrin concentration of 10,000 Monte Carlo simulations of 7 variables (A). The mean, standard deviation, range (B), and the distribution (C) of the simulations, with the experimental end-point estimation of fibrin concentration from the D-dimer ELISA (shown in red). The 7 variables of plasma protein levels and thrombin binding sites were generated uniformly and independently.

**Fig 5:**
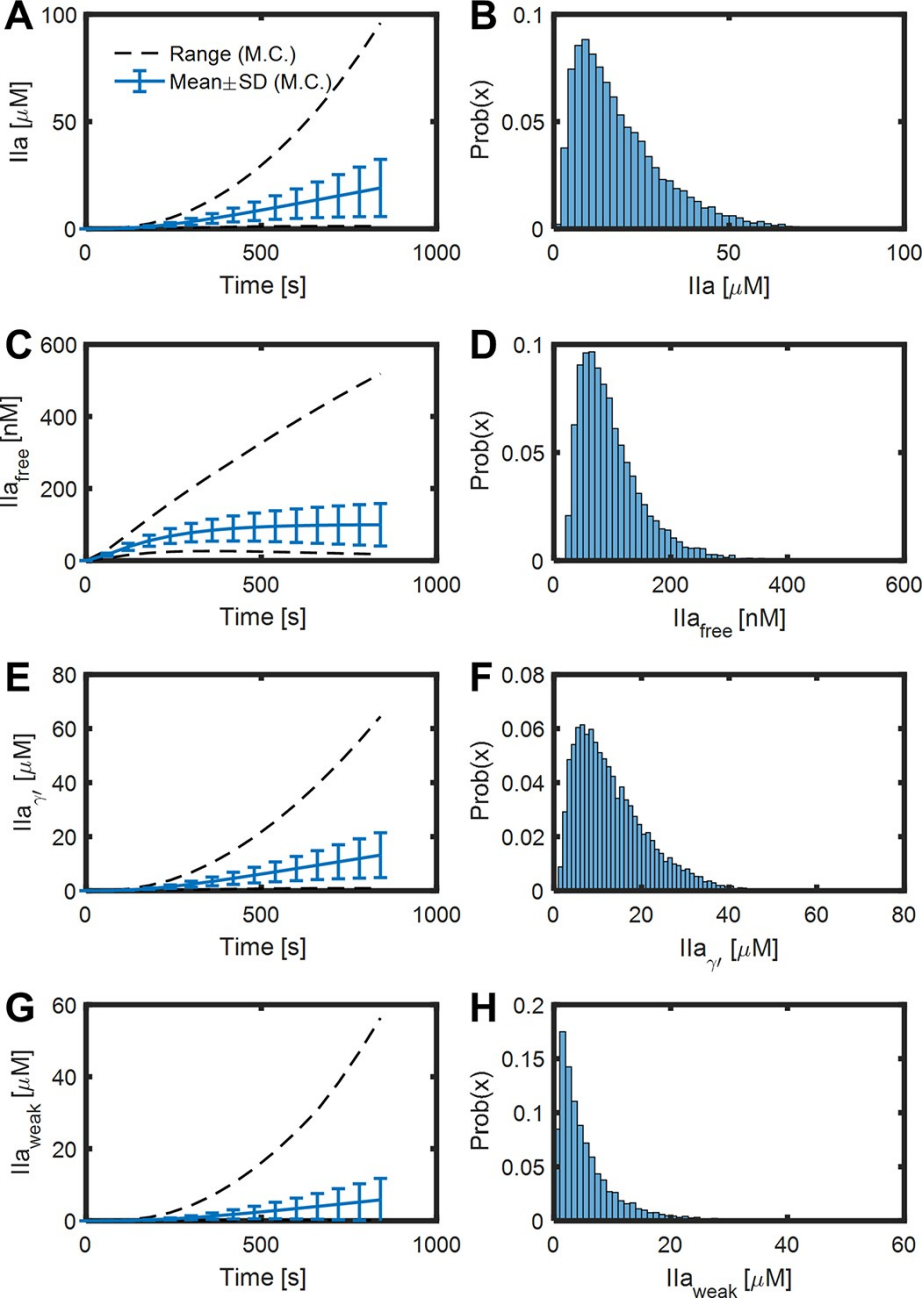
Total, free, and bound thrombin of 10,000 simulations of blood clotting over collagen/ TF under venous flow rate. The mean, standard deviation, range (A, C, E, G) and the distribution at 800 sec (B, D, F, H) of thrombin on different sites of 10000 MC simulation varying plasma protein levels and thrombin binding sites ± 50% uniformly and independently.

**Fig 6:**
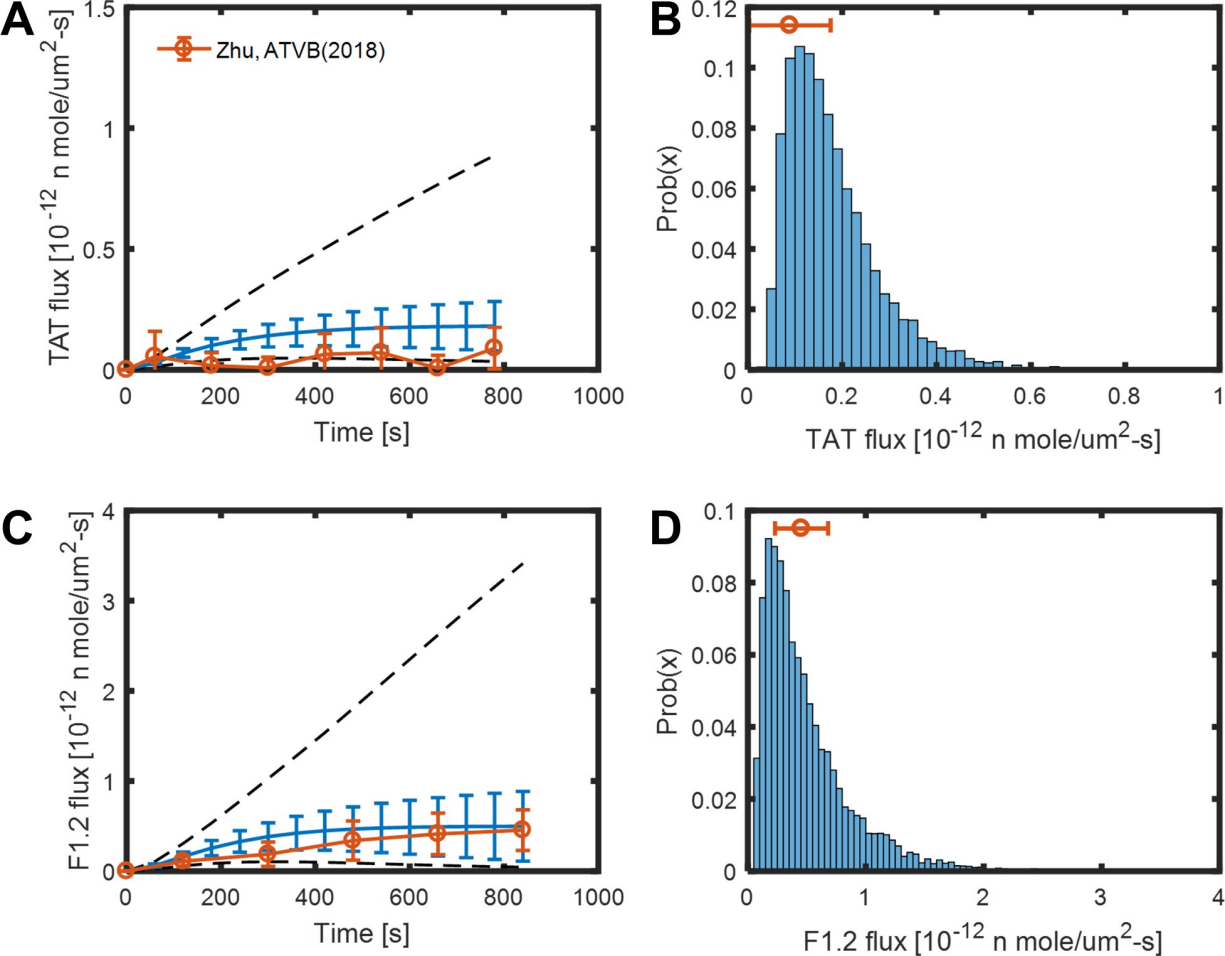
Transient convection-diffusion of thrombin into and out of a fibrin domain exposed to venous flow. The mean, standard deviation, range (A, C) and the distribution at 800 sec (B, D) of TAT and F1.2 flux of 10000 MC simulations varying plasma protein levels and thrombin binding sites ± 50% uniformly and independently. The experimental data of blood clotting over collagen/TF from [19] are shown in red.

To further investigate how the MC sampling of parameters would impact the results of the reduced model, a global sensitivity analysis was performed. A total of 10,000 Monte Carlo simulations were conducted with 7 variables including the levels of 5 plasma protein and 2 thrombin binding sites. Each variable was sampled between 50 and 100% of their baseline uniformly and independently for every simulation. The resulting fibrin concentration for every simulation is shown in **Fig 4A**. The mean, standard deviation and range are shown in **Fig 4B**, and the fibrin concentration distribution at 780 sec are shown in **Fig 4C**. The mean and standard deviation of fibrin concentration are similar to the experimental results, shown in red in **Fig 4A and 4B**, where whole blood was perfused over collagen/TF surface followed by plasmin digestion and D-dimer ELISA [19]. The dynamic results and the distribution at 800 sec of thrombin and other species are shown in **Fig 5** and **S1 File**. Normal range variation of 7 inputs leads to free thrombin ranging most from 20 to 300 nM. The simulation results of TAT and F1.2 Flux are shown in **Fig 6**, and they agree with experimental data (shown in red, [19]) of ELISA analysis of the effluent.

### Conditioned inputs distribution

**Fig 7:**
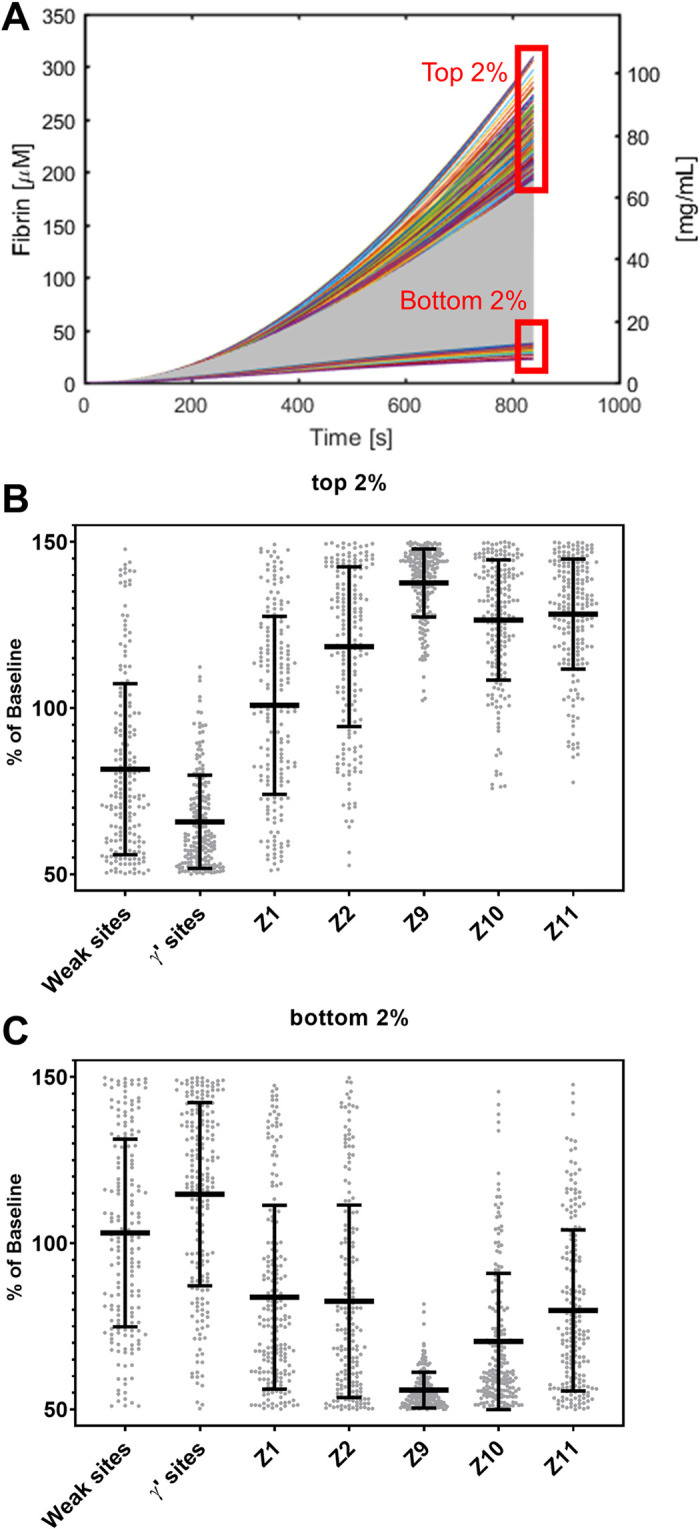
Plasma protein levels and thrombin binding sites distribution of the subsets of top and bottom 2% of fibrin concentration of 10,000 simulations. Fibrin concentration of 10,000 Monte Carlo simulations of 7 variables varying uniformly and independently. Top 2% and bottom 2% are labeled (A). Plasma protein levels and thrombin binding sites distribution of top 2% (B) and bottom 2% of the simulations (C).

We performed 10,000 Monte Carlo simulations by varying 7 variables independently. We further focused on the model’s result on fibrin production and marked the top and bottom 2% of the final fibrin concentration shown in **Fig 7A**. The distribution of the plasma protein levels and thrombin binding sites of top and bottom 2% are shown in **Fig 7B and 7C**. By looking at the subset of top 2% of fibrin generated, the distribution of plasma FIX, FXI levels and gamma’-sites are narrower and skewed away from 100%. These suggest that the high levels of FIX and FXI and less γ’-sites are important and lead to more fibrin, which agreed with the local sensitivity analysis. For the subset of the bottom 2% of fibrin production conditions, low levels of FIX and FX led to less fibrin.

**Fig 8:**
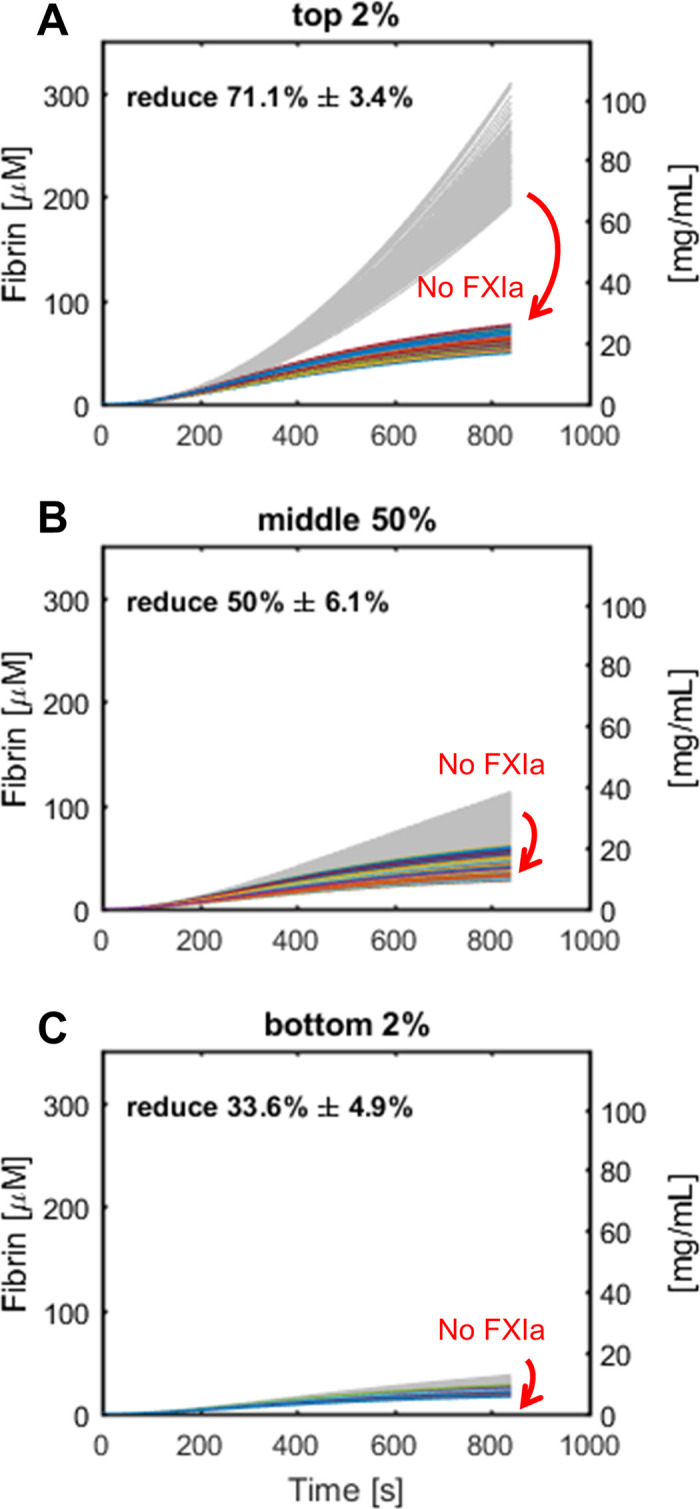
The potency of the blockage of FXIa varies over the subsets of fibrin concentration. The effect of inhibition of FXIa on fibrin concentration of top 2% (A), middle 50% (B), and bottom 2% (C) of 10,000 simulations.

Feedback pathway has emerged as a novel target for antithrombotic pathways with potentially little effects on hemostasis [30] since Factor XI-deficient patients do not have severe hemophilia. Here we evaluated the effect of inhibiting FXIa by turning off the feedback pathway in our simulations. We first labeled the 2%, middle 50% and bottom 2% of the final fibrin concentration in the 10,000 simulations, and then set the FXIa = 0 to see the efficacy of the inhibitor. The results are shown in **Fig 8**. The top 2% of fibrin generators displayed a 71% fibrin reduction by an ideal FXIa inhibitor, whereas the middle 50% and bottom 2% of fibrin generators displayed 50% and 33% fibrin reduction by a perfect FXI inhibitor. Here we showed that the efficacy of feedback pathway inhibitor may vary within a normal range of zymogens.

## Discussion

In this study, we used a reduced coagulation under flow model and performed a sensitivity analysis on the extrinsic pathway of coagulation. We first performed the local sensitivity analysis where each of 5 plasma zymogens and 2 fibrin binding sites for thrombin were varied individually ± 50% from their consensus values. This indicated that the level of FIX and γ’-binding sites are the most influential variables for most of the simulation ensembles. For the global sensitivity analysis, all 7 variables were changed simultaneously and independently from 50 to 150% of the consensus value. With 10,000 Monte Carlo simulations, we showed the distribution of each procoagulants in the model and found that the mean and standard deviation of fibrin generation and TAT and F1.2 flux met with the data measured from healthy donors [19,31]. We also showed that the FXIa inhibitor may have different potency across normal ranged plasma protein and thrombin binding sites, and the top 2% of the final fibrin concentration has the stronger effect of 71% fibrin reduction.

Plasma levels vary among individuals in normal range [11]. Reviews has summarized situations where high levels of coagulation factor elevate thrombotic risk [32]. In a large population-based, case–control study studies, Leiden Thrombophilia Study, research showed that elevated FIX [33] and FXI [34] are related to higher risk for thrombosis. Models has been developed to study the sensitivity to initial clotting factor concentration, both under non-flow [12,35] and under flow [20]. With the reduced model, we highlighted the role of FXI and FIX as risk factors for thrombosis.

Thrombin binds to fibrin clot tightly and localizes on γ’-fibrin [19]. The bindings of thrombin in the clot not only minimize downstream coagulation, but also protects thrombin from antithrombin inhibition [25]. Studies suggested that the variant interacts with other plasma protein and influences on clot formation and strength [36,37]. Fibrinogen γ’ level varies in patients, changes during inflammation and associates with arterial and venous thrombosis clinically [38]. The association between γ’/total fibrinogen ratio and thrombosis remain unclear and somewhat controversial [39]. Some studies suggested that decrease ratio are associated with higher risk for thrombosis [40]. Our reduced ODEs-model demonstrates that low level of γ’-fibrin is the dominant factor in top 2% fibrin generation and it increases thrombin flux and fibrin generation. These results confirm with the explanation that the antithrombin-I activity of fibrin affects coagulation and reduces thrombin and its further activation.

Interestingly, the model predicted a relatively weak sensitivity to fibrinogen levels. High fibrinogen levels are viewed in general as pro-thrombotic, however it is important to recognize that fibrinogen and the γ’-chain variant are acute response genes elevated during inflammation, which may be a complex co-factor for thrombotic risk. Interestingly, some studies have identified elevated fibrinogen as protective, perhaps due to its anti-thrombin-I activity. During the simulations of the model, a 50% reduction in fibrinogen concentration caused only < 10% reduction in fibrin formation, which was unexpected. However, in the multivariable context of the model of diverse initial condition sampling (Fig 7A), there are many situations where low fibrinogen (Z1 in Fig 7C) was occurring with the bottom 2% of simulations producing low fibrin.

Simulations give insights and help experimental design for further discovery in coagulations [41]. Recently, Link et al used the computationally driven approach to identify FV as modifier for hemophilia, further confirm it with experiment, and propose a potential mechanism. Although there are limitations and our model only describes the thrombosis under venous flow over TF-surface, it emphasized crucial reactions which have been often overlooked. With emerging strategies targeting FXI [42], our results provide insights in variation in potency of the inhibitors within normal range of clotting factors. Despite of the simplicity, this reduced model may be useful for its coagulation phenotype and further implementation with multiscale modeling which includes platelet accumulation [4,8,10].

## Supporting information

S1 File Contains the supplement method and figures. (DOCX)
