# Supplementary material for: Sensitivity analysis of a reduced model of thrombosis under flow: Roles of Factor IX, Factor XI, and γ‘-Fibrin
Source: PLoS One. 2021 Nov 23;16(11):e0260366. doi: 10.1371/journal.pone.0260366 (PMC8610249; doi:10.1371/journal.pone.0260366)
Supplement: S1 File — (DOCX) [file pone.0260366.s001.docx]

**Supplement**

**Sensitivity analysis of a reduced model of thrombosis under flow: Roles of Factor IX, Factor XI, and γ’-Fibrin**

**J. Chen, S. L. Diamond***

**^1^** Department of Chemical and Biomolecular Engineering,

Institute for Medicine and Engineering,

University of Pennsylvania,

Philadelphia, PA, United States of America

**Supplement Method**

These 8 ODEs were solved in Matlab R2016b using the ODE solver ode15s*.*

ODE 1.


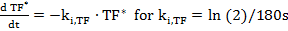

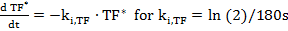


ODE 2.


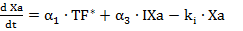

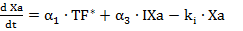


ODE 3.


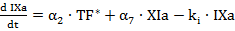

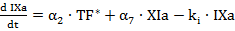


ODE 4.


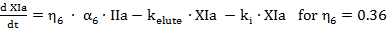

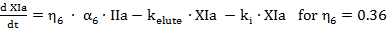


ODE 5.


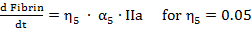

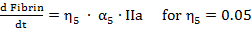


where : and


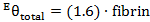

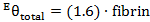

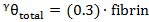

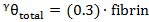


ODE 6.


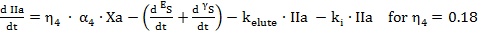

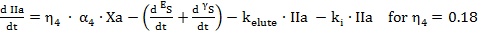


ODE 7.


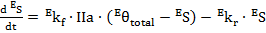

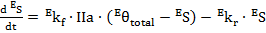


ODE 8.


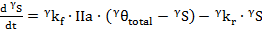

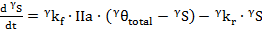


The reduced model for blood clotting on a collagen/TF surface under flow uses 19 parameters, only 3 of which were adjusted to fit the experimental data:

7 kinetic coefficients (α_i_) based on measured kinetics and plasma zymogen levels (**Table 1**)

1 initial surface TF* level based on specified [TF]_o_=1 TF/μm^2^ and [FVIIa]/[FVII] = 0.01.

3 binding parameters: ^E^K_D_ , ^γ^K_D_, k_f_

2 known stoichiometric coefficients: 1.6 E-sites/monomer, 0.3 γ’-sites/monomer

1 elution rate: k_elute_ = ln(2)/2s for free species of thrombin and FXIa

2 inhibition rates: k_i_ = ln(2)/60s for FXa, FIXa, FXIa, FIIa; k_i,TF_=ln(2)/180s for TF*

3 effectiveness factors (η_4_,η_5_,η_6_) ≠ 1, adjusted to fit experimental data.


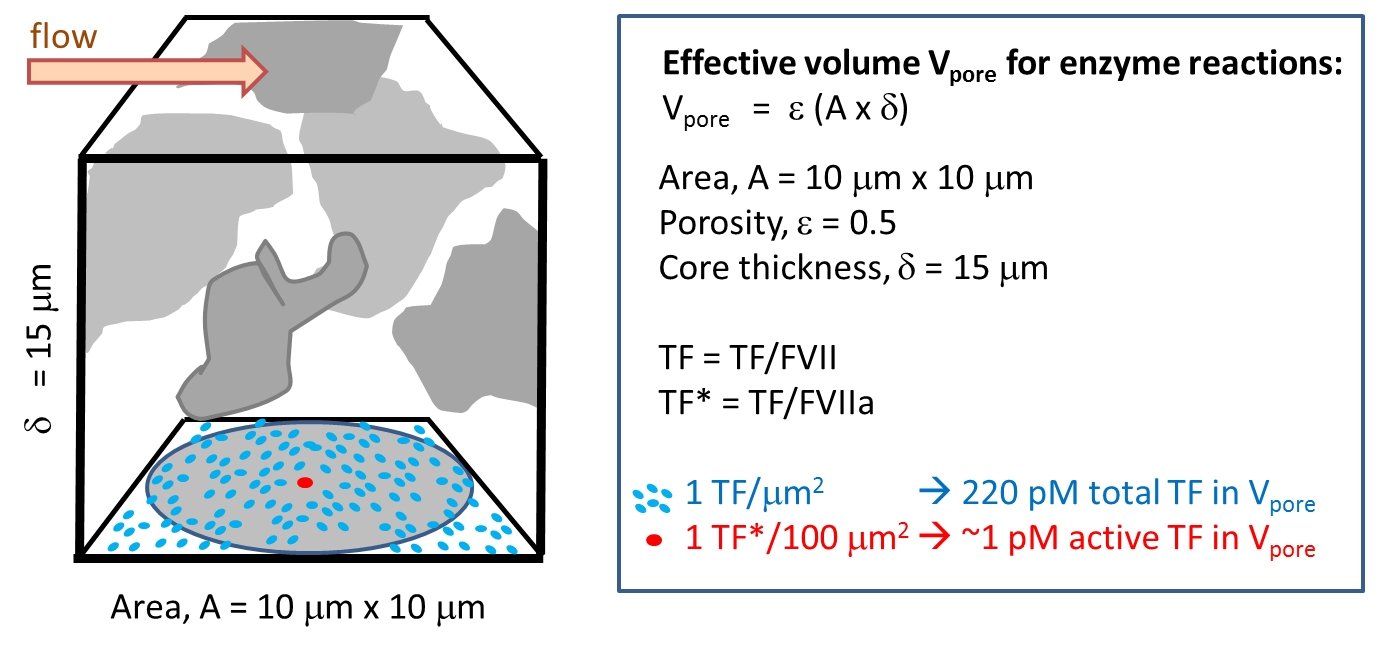


**Supplement Figure 1**. Schematic of the compartment in reduced model. The concentration of active TF* is defined as TF/FVIIa which is homogenized over the porous core volume Vpore. The thrombin core thickness was set to 15-μm, with 50% of platelets by volume. [1]

**Supplement Figure 2**. The results of the reduced model compared to experimental data. Thrombin-antithrombin (TAT) and Fragment F1.2 flux with or without fibrin formation (± GPRP) from whole blood clotting under collagen/TF surface(A,C) and in simulations under identical conditions (B, D). Fibrin intensity dynamics calibrated by end-point D-dimer assay following plasmin degradation (E), while the intrathrombus fibrin concentration was simulated (F). [1]

**Supplement Figure 3.** The concentration of the procoagulants (A, B, C), thrombin distribution (D, E), fibrin, flux of F1.2 and TAT (G,H) with the normal plasma protein levels and thrombin binding sites described in **Table 1**.


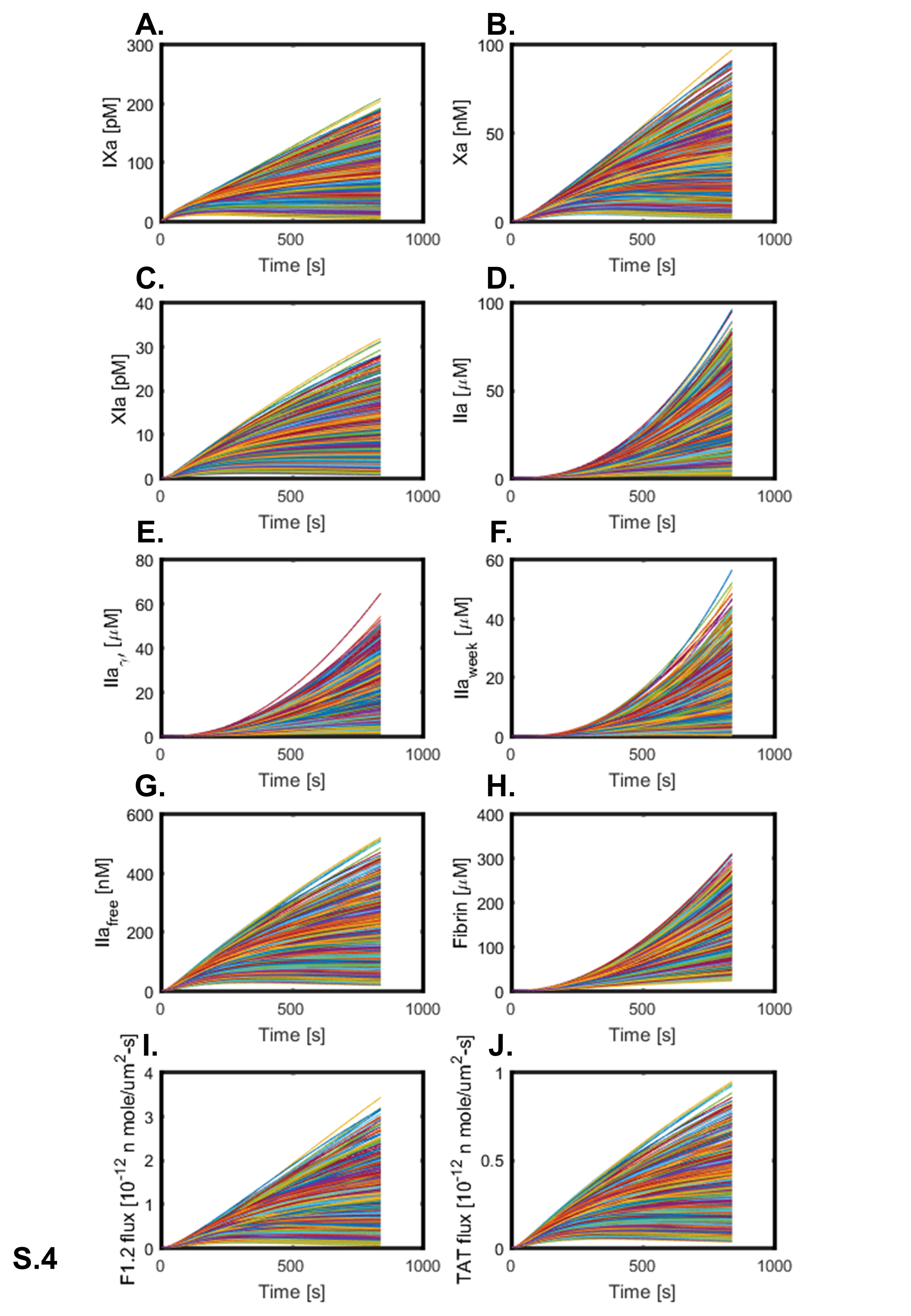


**Supplement Figure 4.** The concentration of the procoagulants (A, B, C), thrombin distribution (D, E, F, G), fibrin (H), flux of F1.2 and TAT (I, J) of 10,000 Monte Carlo simulations of 7 variables. The 7 variables of plasma protein levels and thrombin binding sites were generated uniformly and independently.

**Reference:**

1. Chen J, Diamond SL. Reduced model to predict thrombin and fibrin during thrombosis on collagen/tissue factor under venous flow: Roles of γ’-fibrin and factor XIa. PLOS Comput Biol. 2019;15: e1007266. doi:10.1371/journal.pcbi.1007266

2. Leiderman K, Fogelson AL. Grow with the flow: A spatial-temporal model of platelet deposition and blood coagulation under flow. Math Med Biol. 2011;28: 47–84. doi:10.1093/imammb/dqq005

3. Chatterjee MS, Denney WS, Jing H, Diamond SL. Systems biology of coagulation initiation: Kinetics of thrombin generation in resting and activated human blood. Beard DA, editor. PLoS Comput Biol. 2010;6: e1000950. doi:10.1371/journal.pcbi.1000950

4. Nesheim ME, Tracy RP, Tracy PB, Boskovic DS, Mann KG. Mathematical Simulation of Prothrombinase. Methods Enzymol. 1992;215: 316–328. doi:10.1016/0076-6879(92)15074-M

5. Higgins DL, Lewis SD, Shafer JA, Higginss DL, Lewis SD, Shaferg JA, et al. Steady State Kinetic Parameters for the Thrombin-catalysed Conversion of Human Fibrinogen to Fibrin. J Biol Chem. 1983;258: 9276–9282.

6. Elizondo P, Fogelson AL. A Mathematical Model of Venous Thrombosis Initiation. Biophys J. 2016;111: 2722–2734. doi:10.1016/j.bpj.2016.10.030

7. Haynes LM, Orfeo T, Mann KG, Everse SJ, Brummel-Ziedins KE. Probing the Dynamics of Clot-Bound Thrombin at Venous Shear Rates. Biophys J. 2017;112: 1634–1644. doi:10.1016/j.bpj.2017.03.002
